# Supplementary figures and images for: Influenza A Virus (H1N1) Infection Induces Microglial Activation and Temporal Dysbalance in Glutamatergic Synaptic Transmission
Source: mBio. 2021 Oct 26;12(5):e01776-21. doi: 10.1128/mBio.01776-21 (PMC8546584; doi:10.1128/mBio.01776-21)

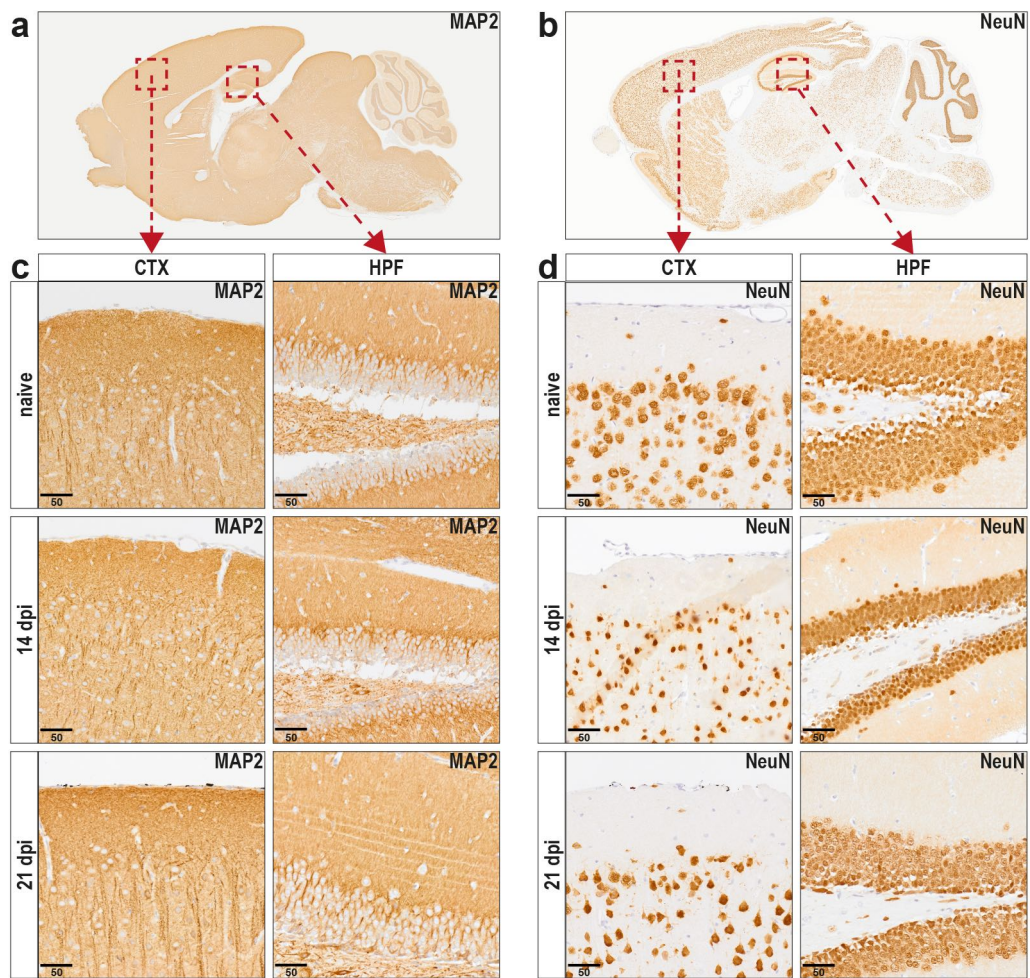

Supplement: FIG S2 [file mbio.01776-21-sf002.pdf]
